# A Review of the Recent Advances in the Pharmacological Management of Parkinson’s Disease

**DOI:** 10.7759/cureus.93123

**Published:** 2025-09-24

**Authors:** Allan Li, Alex Cha, Maria Huang, Sanya Dhami, Sudhakar Pemminati

**Affiliations:** 1 Department of Biomedical Education, California Health Sciences University College of Osteopathic Medicine, Clovis, USA

**Keywords:** leucine-rich repeat kinase 2 (lrrk2) inhibitor, levodopa-carbidopa, parkinson’s disease (pd), tavapadon, dopamine agonist

## Abstract

Parkinson's disease (PD) is a progressive neurodegenerative disorder characterized by the loss of dopaminergic neurons in the substantia nigra, leading to motor and nonmotor symptoms. The pathophysiology of PD involves a complex interplay of genetic, environmental, and biochemical factors, including the accumulation of alpha-synuclein protein aggregates, mitochondrial dysfunction, oxidative stress, and neuroinflammation. Current pharmacological treatments primarily focus on alleviating symptoms, with levodopa (LD) being the gold standard for motor symptom management. Additionally, DOPA decarboxylase (DDC) enzyme inhibitors, dopamine agonists (DA), monoamine oxidase B (MAO-B) inhibitors, catechol-O-methyl transferase (COMT) inhibitors, and centrally acting anticholinergics are employed to modulate dopaminergic/cholinergic signaling and improve quality of life. Despite these advances, the long-term efficacy of existing therapies diminishes over time and presents with severe adverse effects, necessitating the exploration of novel therapeutic approaches. Future drug treatments should aim to address disease progression through neuroprotective strategies, such as gene therapy, immunotherapy targeting alpha-synuclein, and neurorestorative approaches that promote neurogenesis and synaptic plasticity. There are some limitations to consider with these novel therapies, such as current preclinical or early clinical phases with small sample sizes and follow-up trials. This narrative review aims to provide insight into the existing and emerging pharmacological treatment options for the management of PD, while highlighting the need for innovative strategies to improve outcomes for individuals living with PD, comparing the benefits versus risks.

## Introduction and background

Parkinson's disease (PD) is a progressive neurodegenerative disorder of dopaminergic neuron degeneration in the nigrostriatal system; it is multisystemic, involving serotonergic and adenosinergic systems. PD is notable for the aggregation of alpha-synuclein proteins, encoded by the SNCA gene, which form inclusion-filled Lewy bodies and lead to neuronal toxicity and death. This pathology results in hallmark motor symptoms such as resting tremors, rigidity, bradykinesia, and postural instability, primarily attributed to the loss of dopaminergic neurons in the substantia nigra [1,2]. 

**Figure 1:**
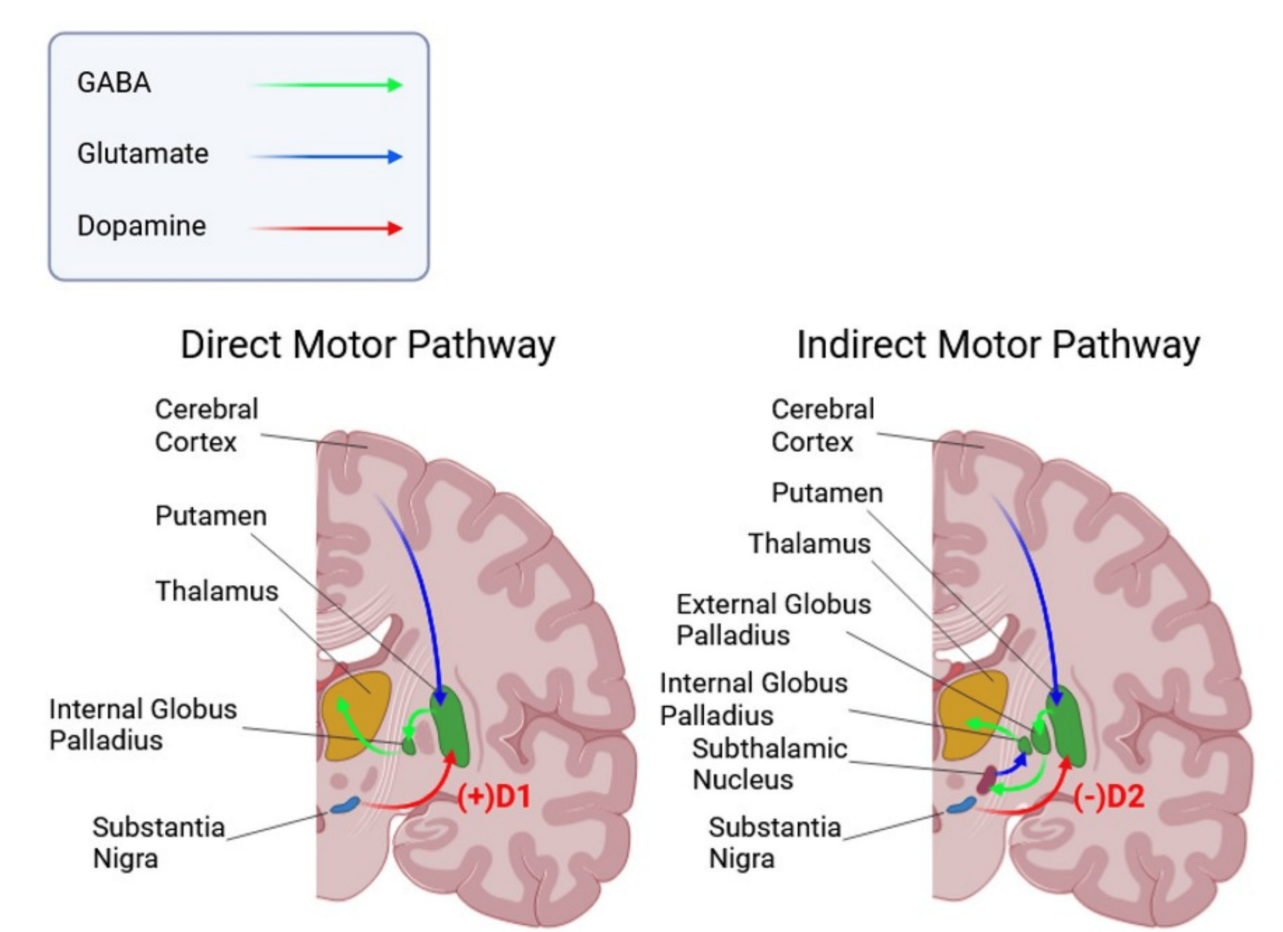
Mechanisms of basal ganglia circuitry disruption in Parkinson’s disease Image credits: Li A, Cha A, Huang M; created in BioRender, (2025) https://BioRender.com/envhdw5

One of the key underlying mechanisms of PD involves dysfunction in the basal ganglia circuitry, a network of interconnected nuclei that modulates motor signals through the direct and indirect pathways, as depicted in Figure 1. In a healthy system, the direct pathway facilitates movement via D1 receptor-mediated excitation, while the indirect pathway inhibits movement through D2 receptor-mediated suppression. Dopaminergic neurons in the substantia nigra pars compacta release dopamine, which binds to D1 receptors in the direct pathway to excite motor-promoting signals and to D2 receptors in the indirect pathway to suppress inhibitory signals [3,4]. Dopamine release is mediated by voltage-gated calcium channels that trigger exocytosis of dopamine-filled vesicles into the synaptic cleft, where dopamine binds to its target receptors. Following synaptic transmission, dopamine is either reabsorbed or degraded by enzymes such as monoamine oxidase B (MAO-B) or catechol-O-methyl transferase (COMT) [5]. This balance between the direct and indirect pathways ensures precise regulation of motor activity by influencing thalamic excitation of the motor cortex.

**Figure 2:**
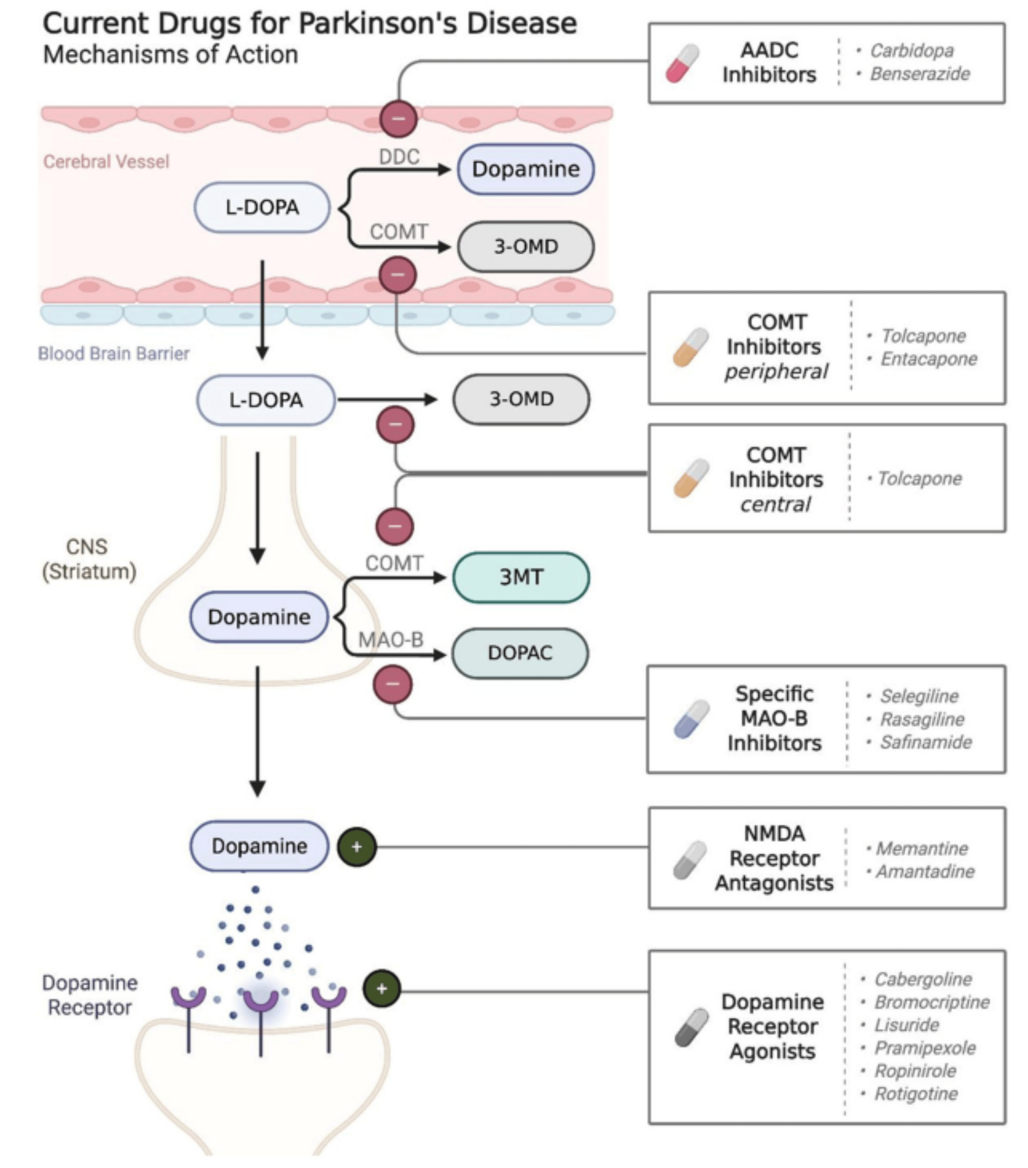
Mechanisms of current Parkinson’s disease medications DDC: dopamine decarboxylase; 3-OMD: 3-O-methyldopa; L-DOPA: levodopa (L-DOPA); 3-MT: 3-methyoxytyramine; DOPAC: 3, 4-dihydroxyphenylacetic acid; COMT: catechol-O-methyltransferase; NMDA: N-methyl-D-aspartate; MAO-B: monoamine oxidase-B (MAO-B) Image credits: Li A, Huang M, Cha A; image created in BioRender (2025), https://BioRender.com/1kfjwux

In PD, progressive degeneration of dopaminergic neurons disrupts this delicate balance, reducing striatal dopamine levels and impairing the direct and indirect pathways. The lack of dopamine-mediated excitation in the direct pathway and the insufficient inhibition of the indirect pathway result in decreased motor cortex activation. The resulting motor dysfunction manifests as bradykinesia, rigidity, and tremors, alongside secondary motor symptoms such as dystonia, dysphagia, and dysarthria. Nonmotor symptoms, including autonomic dysfunction, cognitive decline, and psychological disturbances, further highlight the systemic impact of PD and its pathophysiology [6,7]. Together, these disruptions highlight the fundamental role of dopaminergic signaling in motor control and the impact of its dysregulation in PD. With the disruption of dopaminergic signaling as the main culprit of motor symptoms within PD, pharmacological interventions primarily aim to restore dopamine levels, enhance its action, or modulate related pathways to alleviate motor symptoms. The major drug classes currently used in PD treatment include dopamine precursors, dopamine agonists (DA), DDC inhibitors, COMT inhibitors, anticholinergics, and N-methyl-D-aspartate (NMDA) antagonists [8]. Despite their differing mechanisms of action, these drugs achieve the same central objective: increasing the amount of bioavailable dopamine at the synaptic cleft of dopaminergic transmission sites, as depicted in Figure 2.

## Review

Methods

This literature review on current and novel therapies for PD was initiated on May 22, 2024. Relevant studies were identified through a comprehensive search, primarily of PubMed/MEDLINE (Medical Retrieval Analysis and Retrieval System Online), Embase, Scopus, and other methods using the following search terms: "Parkinson's disease AND (pharmacotherapy OR therapeutics OR treatments OR drugs OR medication OR intervention OR therapy OR neuroprotection OR disease-modifying OR symptomatic treatment OR novel therapy OR repurposed drugs OR clinical trials OR experimental treatment OR adjunct therapy OR dopamine replacement OR neurorestoration OR neuroinflammation OR gene therapy OR targeted therapy OR small molecules OR biologics OR immunotherapy OR regenerative medicine OR gut microbiome OR drug delivery OR combination therapy OR non-dopaminergic therapy)."

**Figure 3:**
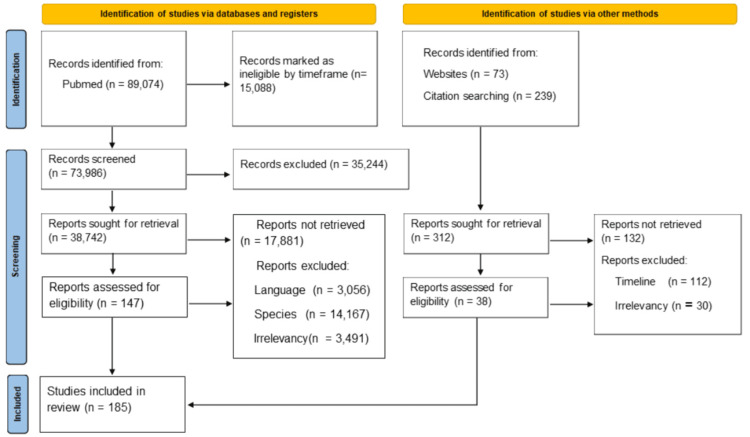
The PRISMA flow diagram depicting the study selection process PRISMA: Preferred Reporting Items for Systematic Reviews and Meta-Analyses

The search was limited to articles published from 2000 to 2025 to ensure the inclusion of the most recent advances in PD treatment. The last search date was August 2, 2025. At this stage, drugs and drug categories were manually selected to capture any additional publications or trials that might have been missed by the electronic search, while excluding any articles that were not suitable or pertinent to the systematic review (Figure 3). The Preferred Reporting Items for Systematic Reviews and Meta-Analyses (PRISMA) guidelines were followed to ensure a vigorous and transparent search process, aiming to minimize bias and maximize the retrieval of pertinent studies for review [9]. Information regarding current clinical trials was obtained through a detailed search of clinicaltrials.gov for specific drugs of interest that we identified above. This search was limited to phases 1, 2, and 3 trials and covered only trials that are currently recruiting or ongoing.

Inclusion criteria required that all studies be published in English and focus on therapeutic interventions for PD. Duplicate and non-accessible studies were excluded to maintain the integrity of the review. The selection process involved screening for relevance to both established pharmacotherapies and emerging treatments for PD, with an emphasis on those offering disease-modifying potential or novel mechanisms of action. Ultimately, 185 studies met our stringent criteria and were included in the review. 

Current therapies

Dopamine Precursors

Dopamine precursors serve as the mainstay of PD treatment by directly addressing the loss of dopaminergic neurons that disrupt basal ganglia circuitry. As dopamine levels decline, the balance between the direct and indirect pathways is impaired, leading to hallmark motor symptoms such as bradykinesia, rigidity, and tremors [6,7,10]. Dopamine precursors, such as levodopa (LD), can cross the blood-brain barrier and be converted into dopamine, effectively replenishing striatal dopamine stores and restoring basal ganglia function [11]. By enhancing excitation of the direct pathway and inhibition of the indirect pathway, LD offers substantial restoration of motor control, cementing its role as the cornerstone of PD symptom management.

Levodopa: LD, a dopamine precursor, is the primary and most studied pharmacological treatment currently available for PD. The discovery of LD as an effective treatment for PD was driven by early research on reserpine in the 1940s and 1950s, which induced Parkinsonism by depleting dopamine levels. This led Swedish pharmacologist Arvid Carlsson to identify dopamine’s key role in motor control in 1957, a discovery that later earned him the Nobel Prize in Medicine in 2000. Building on this work, Viennese pharmacologist Oleh Hornykiewicz demonstrated selective striatal dopamine depletion in PD brains, prompting the first clinical administration of intravenous LD in 1961, which produced dramatic motor improvements. However, widespread clinical adoption had to wait till Cotzias’ landmark 1967 study, which demonstrated LD’s potent symptomatic relief when administered in high oral doses, establishing it as the gold standard PD therapy. Further advancements in the 1970s, including the introduction of DDC inhibitors such as carbidopa (CD) and benserazide, significantly enhanced LD’s bioavailability and reduced peripheral side effects. This led to the development of Sinemet, the first commercial LD/CD therapy [12].

LD functions by replenishing dopamine stores in the brain, reversing the PD symptoms that result from an imbalance of neurotransmitters. LD is converted to dopamine through the action of the dopa decarboxylase (DDC) enzyme. There are two main enzymatic pathways responsible for the metabolism of LD, DDC, and COMT. Peripheral circulation of dopamine has been known to lead to such side effects as nausea, vomiting, and hypertension [13]. LD can be given orally, via oral inhalation, continuous intestinal infusion, or LD/CD intestinal gel via nasojejunal or percutaneous gastrojejunostomy tube. Thus, LD is often administered in combination with a DDC inhibitor to reduce the conversion of LD to dopamine outside the brain [11]. LD/CD remains the gold standard for management of the motor symptoms of PD. Unfortunately, long-term treatment with LD leads to unavoidable extrapyramidal side effects after several years [14]. As a result, most registered clinical trials aim to extend the therapeutic window of LD/CD while mitigating adverse events. These can be classified as LD/CD derivative add-on therapies [15].

DDC Inhibitors

DDC inhibitors act by preventing the metabolism of LD in the periphery, thus reducing LD-related peripheral adverse effects, such as nausea, vomiting, and cardiovascular disturbances [16]. Furthermore, the administration of DDC inhibitors has been shown to demonstrate up to a 10-fold increase in CNS bioavailability of LD [17]. Recent studies have even suggested that levels of DDC found in CSF can accurately identify preclinical patients progressing to clinical Lewy body dementia (p = 0.035) and are not yet clinically validated [18]. As Lewy body dementia is closely associated with PD, this is a potential research target for early identification of PD [19].

Carbidopa: CD is the standard DDC inhibitor given in conjunction with LD during PD treatment. By reducing the peripheral metabolism of LD, CD can mitigate peripheral side effects such as nausea, vomiting, and LD-induced motor dyskinesia [11]. A randomized, double-blind study demonstrated that increasing the dosage of CD in combination with LD and entacapone significantly improved 'off' time, the period when medication effects diminish, and symptoms worsen. The most notable improvements were observed with the highest tested dose of CD (105 mg), showing statistical significance (p = 0.006) [20]. CD itself is a safe medication, as a randomized, double-blind clinical study by Brod et al. found that a sixfold increase compared to the normal dosage did not result in any increase in side effects, while also exhibiting a small increase in bioavailability of LD (p^2 ^= 0.014) [21]. These findings are consistent with a wealth of evidence supporting the long-term efficacy and safety of LD/CD treatments in reducing “off” time and improving the quality of life in patients with PD [22].

Benserazide: Benserazide is another DDC inhibitor that can be given in combination with LD. The mechanism of action and therapeutic effects are very similar to CD. A study from Iwaki et al. demonstrated no significant difference in pharmacokinetics between LD/CD and LD/benserazide administration [23]. Furthermore, an analysis of three randomized, double-blind, six-month, phase III studies showed no statistical difference in motor symptom improvement [24]. A later study by Baba et. al would compare LD/CD 100/10mg with LD/benserazide 100/25 mg, the only two medications available on the Japanese market. In that study, the researchers found that LD/benserazide has a shorter mean duration to the emergence of unwanted motor fluctuations than does LD/CD (3.1 ± 1.2 vs 5.0 ± 1.4 years, p<0.01) [25]. The findings are summarized in Table 1.

**Table 1:** Dopamine modulators in the management of Parkinson's disease CD: carbidopa; DDC: dopamine decarboxylase; LD: levodopa; GERD: gastroesophageal reflux disorder; Cmax: maximum plasma concentration

Study; drug	Study population	Mechanism of action	Benefits (B) and risks (R)
Trenkwalder et al., 2019, USA [20]; carbidopa/levodopa/entacapone	N = 117	Dopamine replacement and metabolism modulation	B: improvement of “off” time with an increase in CD dosage administered. R: nausea, dizziness, drug-effect decrease, dyskinesia
Brod et al., 2012, USA [21]; carbidopa	N = 12	DDC inhibitor	B: increased bioavailability of LD. R: pain, GERD, back injuries, sciatica
Iwaki et al., 2014, Japan [23]; levodopa/carbidopa and levodopa/benserazide	N = 48	DDC inhibitor	B: higher plasma LD levels with benserazide. Comparable pharmacokinetics for patients and healthy subjects. R: variability in drug clearance
Kuoppamäki et al., 2015, Finland [24]; levodopa/carbidopa and levodopa/benserazide	N = 551	DDC inhibitor	B: increased efficacy and treatment effects when compared to placebo. R: dyskinesia, diarrhea, hyperkinesia, nausea, constipation, hypokinesia
Baba et al., 2022, Japan [25]; levodopa/carbidopa and levodopa/benserazide	N = 52	DDC inhibitor	B: LD/benserazide reaches C_max_ quicker than LD/CD. More useful for patients requiring immediate relief from motor symptoms. R: earlier onset of motor fluctuations and “wearing-off” phenomenon for LD/benserazide combination therapy

Catechol-O-methyltransferase (COMT) Inhibitors

Application of DDC inhibitors with LD therapy has been found to lead to increased activity of the COMT pathway. As a result, COMT inhibitors are often used to further prevent LD breakdown in the periphery. COMT inhibitors prevent the metabolism of LD to 3-O-methyldopa [26]. While 3-O-methyldopa has not been found to affect the action of LD in PD, COMT inhibitors have been found to increase the therapeutic response in PD patients [27]. Thus, COMT inhibitors (entacapone, opicapone, and tolcapone) are often paired as adjuncts with LD and DDC during treatments.

Entacapone: Entacapone acts peripherally and does not cross the blood-brain barrier. It increases the ON time and reduces the OFF time by one to two hours in PD patients experiencing wearing-off symptoms, allowing for a reduction in daily LD dose. While some studies show that there is no significant improvement in the Unified Parkinson’s Disease Rating Scale (UPDRS) score compared with patients taking only LD, improvement in quality-of-life measures is noted [28]. Furthermore, entacapone has a short half-life of one hour, corresponding well with LD’s half-life. As a result, it can easily be combined with LD/CD in one triple combination tablet known commercially as Stavelo [16]. However, a meta-analysis by Liao et al. showed that treatment with LD/CD/entacapone had statistically significant adverse effects compared to controls (p<0.00001) [29].

Tolcapone: While effective, tolcapone is not widely used due to potential liver toxicity, having been implicated in elevated liver enzymes and liver failure in some patients [30]. Furthermore, its longer half-life limits its administration to three times daily, irrespective of LD dosing, increasing the complexity of dosing and potentially decreasing compliance [31]. However, in contrast to entacapone, which exhibits biphasic elimination, tolcapone has a single elimination half-life of two to three hours [32]. Furthermore, some animal studies have shown that tolcapone can penetrate the blood-brain barrier, which may potentially exert some central effects [33]. Recent studies suggest that the therapeutic benefits may outweigh potential adverse effects through close monitoring of hepatic function to mitigate liver failure [34]. The findings are summarized in Table 2.

**Table 2:** Catechol O-methyltransferase inhibitors in the management of Parkinson's disease COMT: catechol-O-methyltransferase; PDQ-39: Parkinson's Disease Questionnaire 39; SF-36: 36-item Short-Form Health Survey; PSI: Parkinson’s Symptom Inventory; UPDRS: Unified Parkinson’s Disease Rating Scale; LD: levodopa

Study; drug	Study population	Mechanism of action	Benefits (B) and risks (R)
Olanow et al., 2004, USA [28]; entacapone:	N = 555	COMT inhibitor (peripheral)	B: significant improvement of PDQ-39, SF-36, and PSI. R: no improvement in UPDRS score compared with patients taking only LD, increased frequency of nausea and dyskinesia
Liao et al., 2020, China [29]; levodopa/carbidopa/entacapone	N = 1983	Dopamine replacement and metabolism modulation	B: improves UPDRS Part I nonmotor and UPDRS Part II motor symptoms affecting daily activity. R: dyskinesia, urine discoloration, nausea, diarrhea, dizziness, abdominal pain
Borges, 2005, Portugal [30]; tolcapone	N = 3	COMT inhibitor (peripheral and central)	B: improvement in symptoms and quality of life in PD patients. R: hepatic failure
Artusi et al., 2021, Italy [34]; tolcapone	N = 4780	COMT inhibitor (peripheral and central)	B: average improvement of 3.6 points on UPDRS Part III. Decreased LD dosage requirement. R: elevated liver enzyme, potentially severe liver injury, dyskinesia, nausea, insomnia, anorexia, dystonia, diarrhea, orthostatic symptoms, excessive dreaming

Dopamine Agonists (DA)

DAs are a cornerstone in the pharmacological management of PD. These agents mimic the effects of dopamine by directly stimulating dopamine receptors. They are divided into two main categories: ergot-derived and non-ergot-derived agonists [35]. The first-generation, ergot-derived drugs, such as bromocriptine, pergolide, and cabergoline, have been associated with adverse effects such as heart failure [36]. As a result, they have largely fallen out of favor and are rarely prescribed.

In contrast, second-generation non-ergot agonists, such as pramipexole, ropinirole, and rotigotine, are more selective for dopamine D2 and D3 receptors and are better tolerated. These “cleaner” agents effectively alleviate the motor symptoms of PD while also reducing the risk of fibrotic side effects [37]. Unfortunately, second-generation non-ergot agonists have a shorter half-life and must be taken multiple times a day, which may affect patient compliance [38]. DAs are especially important in the early stages of PD as they delay the need for LD while also helping to manage motor fluctuations in advanced disease.

Bromocriptine: Bromocriptine is one of the earliest DAs introduced for the treatment of PD. It is an ergot-derived medication that directly stimulates dopamine D2 receptors, which helps to compensate for the progressive dopamine deficiency characteristic of PD. However, recent studies have highlighted a lack of evidence to support the efficacy of bromocriptine in early PD [39]. In fact, prior clinical research on the efficacy of bromocriptine on PD research has major methodological problems and extreme variability in measurement scales [40]. A 2023 network meta-analysis further supported these concerns by showing that bromocriptine had the lowest efficacy among DAs in improving motor function (as measured by UPDRS Part II, UPDRS Part III, and combined scores), and it ranked last in overall tolerability and withdrawals due to adverse events [41]. Although bromocriptine was associated with the lowest incidence of dyskinesia (OR = 0.05, 95% CI = 0.01-0.15) compared to LD, its high rates of treatment discontinuation and inferior symptomatic control significantly limit its utility. As an ergot-derived DA, bromocriptine has also been associated with fibrotic reactions at the heart, lungs, and retroperitoneal space [42]. Lastly, bromocriptine withdrawal has also been found to be associated with neuroleptic malignant syndrome [43]. As a result of all these concerns, bromocriptine is no longer regularly prescribed for the treatment of PD.

Cabergoline: Cabergoline is another ergot-derived DA used in the treatment of PD and other dopaminergic disorders. Like bromocriptine, it also works through selective stimulation of dopamine D2 receptors, making up for dopaminergic deficiency in PD. Cabergoline has a very long half-life and is effective when given once daily [44]. Cabergoline is effective in treating motor symptoms and improving activity of daily life scores [45]. In a five-year prospective randomized study involving 98 Japanese patients, adverse events associated with the initial treatment using cabergoline were documented, including edema, gastrointestinal disorders, malignant neoplasms, cardiac valvular regurgitation, and depression [46]. The study compared the effectiveness of cabergoline and LD in reducing motor complications. Although cabergoline was associated with a 43% lower risk of motor complications compared to LD, this difference did not reach statistical significance (HR = 0.57, p = 0.347).

As an ergot-derived DA, cabergoline, like bromocriptine, is also associated with fibrotic complications of the heart, lungs, and retroperitoneal space [42,47]. As a result, cabergoline is now less commonly prescribed, with preference given to second-generation non-ergot DAs, which will be discussed later in this paper. Despite its reduced usage, cabergoline is still an effective option for patients when non-ergot agonists are unsuitable. Regular monitoring of cardiac and pulmonary function can further limit the risk of fibrotic complications in patients taking cabergoline.

Lisuride: Lisuride, an ergot-derived DA primarily targeting D2 receptors, is used for both early and advanced PD as well as migraine prevention. Although it is not available in the U.S. market, lisuride remains in use in other countries due to its unique pharmacological profile as a potent D2 agonist, partial D1 agonist, and 5-HT1A agonist -enabling both anti-Parkinsonian and anti-dyskinetic effects [48]. In a 2002 four-year, prospective, randomized, open-label trial involving 40 patients with advanced PD, lisuride infusion therapy was compared to oral LD treatment. Lisuride therapy led to a 59.3% improvement in daily ‘OFF’ time (p<0.0001), whereas LD treatment resulted in a 21.4% increase in ‘OFF’ time. Additionally, dyskinesia scores improved by 49% in the lisuride group but worsened by 59% in the LD group (p<0.0001), underscoring the superior efficacy of lisuride in reducing both motor fluctuations and dyskinesia severity [49]. 

A subsequent long-term follow-up study involving 82 newly diagnosed, idiopathic PD patients, participants were randomized into two treatment groups comparing the outcomes of classic single-drug therapy using LD against combination therapy involving LD and lisuride. Over five years, the combination therapy demonstrated significant advantages in improving activities of daily living (UPDRS Part II) and motor function (UPDRS Part III), with superior outcomes observed as early as the first few months and maintained throughout the study (p<0.01). Additionally, the combination group experienced fewer motor complications, such as dyskinesia and fluctuations, as indicated by the UPDRS Part IV addendum (p<0.05), while requiring a significantly lower LD dose at 60 months (387.5 mg/day vs. 446.7 mg/day, p<0.01). These findings highlight the long-term efficacy and symptom-stabilizing effects of combining LD with lisuride in recently diagnosed Parkinson’s patients [50].

Lisuride is not only the second-oldest and most potent ergot-derived DA [48], but also the only one with strong 5-HT₂B antagonistic activity, reducing the risk of cardiac valvulopathy and fibrosis commonly associated with cabergoline [51]. However, despite the marked benefits, side effects such as nausea, vomiting, and orthostatic hypotension due to dopaminergic system activation remain, though they can be managed with dopamine antagonists.

Pramipexole: Pramipexole is a non-ergot-derived dopamine D2/D3 receptor agonist that was approved in 1997 for use either as monotherapy or in combination with first-line treatments in PD [52]. It is particularly beneficial for younger PD patients who often experience more motor symptom fluctuations when on an LD/CD regimen [53]. Pramipexole has demonstrated efficacy as both a monotherapy in early PD and an adjunctive therapy in advanced disease. It provides additional benefits in alleviating depressive symptoms commonly seen in PD patients. While generally well-tolerated, pramipexole is associated with a higher rate of dopaminergic adverse effects when compared to LD treatment [54].

One advantage of using pramipexole is the ability to lower the dosage of LD/CD, which can help reduce “OFF” time symptoms [53]. The extended-release (ER) formulation of pramipexole offers a once-daily treatment option for idiopathic PD. It provides similar exposure over 24 hours as the three-times-daily immediate-release (IR) formulation but with fewer fluctuations in plasma pramipexole concentrations. In clinical trials, pramipexole ER significantly improved PD symptoms, as measured by the UPDRS Part II and UPDRS Part III, and reduced off-time in advanced disease patients. The ER formulation was generally well-tolerated, with adverse events occurring at rates like pramipexole IR [55].

However, pramipexole is associated with an increased risk of certain adverse effects, particularly hallucinations. A study found that pramipexole was associated with a higher risk of hallucinations compared to ropinirole when compared with placebo (RR 5.2 [95% CI, 1.97-13.72]). There was no significant difference in the risk of dizziness, nausea, or hypotension between pramipexole and LD. The pooled relative risk of somnolence with pramipexole compared to placebo was 2.01 (95% CI, 2.17-3.16), indicating a higher risk of somnolence. Overall, compared with placebo, pramipexole was associated with a higher risk of hallucinations, while ropinirole carried a greater risk of somnolence and hypotension [56].

Ropinirole: Ropinirole is a non-ergoline DA with selective affinity for dopamine D2-like receptors, primarily targeting motor-related pathways in PD. It has minimal affinity for non-dopaminergic brain receptors [57]. This drug is currently available in two oral formulations: immediate-release tablets (0.25 to 5 mg) taken three times daily and prolonged/extended-release tablets (2 to 12 mg) taken once daily [58]. Compared to LD, ropinirole is associated with higher rates of somnolence, hallucinations, and lower extremity edema, with warnings on the label for risks such as sudden sleep attacks and impulse control disorders (ICDs), including compulsive behaviors like gambling and binge eating [59].

In 2020, a randomized, double-blind, parallel-group, placebo-controlled trial evaluated the efficacy and safety of ropinirole hydrochloride patch compared to placebo and extended-release tablets in PD patients using LD. The least squares mean change in UPDRS Part III total score was -9.8 with the ropinirole patch, -4.3 with placebo, and -10.1 with the ropinirole tablet. The difference between the ropinirole patch and placebo was -5.4 (p<0.0001), demonstrating superiority of the patch over placebo. Additionally, the difference between the patch and tablet groups was 0.3, with the upper limit of the 95% confidence interval smaller than the noninferiority limit of 2.5 (p<0.0001). The main adverse effects reported were application site erythema and pruritus, neither of which was severe enough to initiate termination of treatment [60]. These results provide evidence that patients could be offered an alternative treatment option for ropinirole through a dermal patch, which offers the benefits of convenience and ease of use.

Rotigotine: Rotigotine is a non-ergolinic DA that binds to dopamine receptors D1 through D5, with significantly higher affinity for D2 and D3 receptors compared to dopamine [61]. It is delivered via a once-daily transdermal patch that provides continuous drug release for over 24 hours. In the EU, it is approved as monotherapy for early PD and as combination therapy with LD for all stages of the disease [62]. Rotigotine was withdrawn from the market in 2008 after the FDA suspended its marketing authorization due to crystal formation in certain patches, which could compromise bioavailability and effectiveness. Following a reformulation to resolve these issues, the drug was reintroduced in the U.S. in 2012, with numerous studies since conducted to evaluate its efficacy [63].

The rotigotine transdermal patch has demonstrated clinical efficacy in early-stage PD with a tolerability profile comparable to other non-ergot DAs. It provides continuous 24-hour drug release via once-daily application, offering consistent symptom control. A multicenter, double-blind study compared the safety and efficacy of transdermal rotigotine with placebo in early-stage PD patients treated with rotigotine, showing a significantly greater improvement in UPDRS scores (mean difference of 5.28 ± 1.18, p<0.0001) and higher responder rates (48% vs. 19%, p<0.0001). The greatest improvements were seen in motor symptoms (UPDRS Part III), with a mean change of −3.50 ± 7.26. Adverse events associated with rotigotine occurred with more frequency compared to the placebo, including application site reactions (44% vs. 12%), nausea (41% vs. 17%), somnolence (33% vs. 20%), and dizziness (19% vs. 13%) [64].

Two phase 3 studies had demonstrated that rotigotine (≤8 mg/24 h) was effective and well-tolerated in early-stage PD in mostly Caucasian patients, so a phase 3 study evaluated its efficacy in Chinese patients with early-stage PD. In this study, rotigotine significantly improved UPDRS Part II + III total scores compared to placebo (LSmean difference: -4.82 [-7.18 to -2.45]; p<0.0001), with higher responder rates (42.3% vs. 22.3%; P = 0.0006) and significant improvements in UPDRS Part II and UPDRS Part III (motor function) subscores (p<0.0005). In this study, adverse event rates are presented as the incidence in the rotigotine group compared to the placebo group. The adverse event profile in the Chinese cohort was consistent with prior studies, with common side effects including nausea (8.9% vs. 3.3%), dizziness (8.1% vs. 5.7%), pruritus (8.1% vs. 4.1%), somnolence (8.1% vs. 3.3%), erythema (6.5% vs. 1.6%), and vomiting (5.6% vs. 1.6%). A total of 5.3% of patients discontinued treatment due to adverse events. Despite these effects, rotigotine was well tolerated and effective in improving both motor and non-motor symptoms in Chinese patients, with efficacy consistent across ethnic groups [65].

These findings have contributed to rotigotine's recognition as a globally accepted treatment for PD [7]. The findings are summarized in Table 3. Impulse control disorders (ICD) are potential behavioral adverse effects like problematic or pathological gambling, compulsive buying, hypersexuality, and binge eating; about 14-17% of patients treated with dopamine agonists develop at least one ICD, compared to 6-7% of those not exposed to dopamine agonists [66] 

**Table 3:** Dopamine receptor agonist in the management of Parkinson’s disease 5-HT₂B: serotonin (5-hydroxytryptamine 2B) receptor; PD: Parkinson’s disease

Study; drug	Study population	Mechanism of action	Benefits (B) and risks (R)
Montastruc et al., 2017, France [36]; dopamine agonists	N = 16,897	Dopamine agonists	R: heart failure
Zhang et al., 2023, worldwide [41]; bromocriptine	N = 2112	Ergot-derived D2 receptor agonist with partial D1 antagonist activity	B: alleviation of PD symptoms, lower incidence of dyskinesia. R: poor tolerability, lower efficacy, somnolence, nausea, postural hypotension, dizziness, headache, and insomnia
Utsumi, 2012, Japan [46]; cabergoline	N = 98	Long-acting ergot-derived D2 receptor stimulator	R: motor complications, dyskinesia, edema, GI disorder, malignant neoplasms, depression
Stocchi et al., 2002, Italy [49]; lisuride	N = 40	Ergot-derived D2 receptor agonist with 5-HT₂B antagonism	B: significant reduction of motor fluctuations and dyskinesia. R: nausea, psychiatric complications, hypotension, hypersexuality, skin nodules
Hattori et al., 2020, Japan [60]; ropinirole	N = 428	Non-ergot selective D2/D3 receptor agonist	B: improved motor function, reduction in “off” time, stable blood drug concentration. R: application-site erythema, application-site pruritus, somnolence, nausea, dyskinesia, hallucinations
Zhou et al., 2013, China [63]; rotigotine	N = 247	Non-ergot D1/D2/D3 receptor agonist	B: improvement of motor function. R: nausea, dizziness, pruritus, somnolence, erythema, vomiting

Monoamine oxidase-B inhibitors

MAO-B inhibitors, such as selegiline, rasagiline, and safinamide, inhibit the activity of the MAO-B enzyme, which is responsible for breaking down dopamine in the brain. By reducing dopamine metabolism, MAO-B inhibitors increase the availability of dopamine in the brain and alleviate the motor symptoms associated with dopaminergic deficiency in PD [67]. MAO-B inhibitors are especially effective in the early stages of PD, where they can provide symptomatic relief as a monotherapy while also delaying the need for LD; they are also able to delay the onset of unavoidable extrapyramidal side-effects associated with LD usage [68]. MAO-B inhibitors are generally well-tolerated, but common side effects can include orthostatic hypotension, dizziness, drowsiness, insomnia, and nausea [69]. Another important characteristic of MAO-B inhibitors is their ability to, when taken in large concentrations, also inhibit MAO-A, which is responsible for breaking down neurotransmitters. Consequently, when large amounts of tyramine-rich foods are consumed, this can lead to overstimulation of postsynaptic adrenergic receptors and may result in life-threatening blood pressure elevation [70]. However, its relatively favorable safety profile and neuroprotective potential have made MAO-B inhibitors an extremely important component of PD management strategies today.

Selegiline: Selegiline is an irreversible inhibitor of MAO-B. The first clinical trials of selegiline were conducted in 1987, known as the DATATOP clinical trial. This study showed that selegiline significantly increased the time until LD was needed as compared to placebo [71]. Another phase 3 study conducted in Japan on 292 subjects showed that administration of selegiline as a monotherapy improved Parkinsonian symptoms significantly when compared to placebo (p = 0.0005). Furthermore, the safety profile of selegiline was comparable to placebo with no significant difference in the number of adverse events (p>0.05) [72]. However, the effect of selegiline appears to be predominantly symptomatic instead of disease-modifying; selegiline improves symptoms rapidly, but its effects appear to diminish over time [73]. As a result, the delay until initiation of LD may be because selegiline itself has mild symptomatic effects that improve motor symptoms in PD, instead of being neuroprotective [74]. Some side effects of selegiline that have been identified include sudden sleep episodes, orthostatic hypotension, arrhythmia, mental status alteration, hallucination, extrapyramidal symptoms, dyskinesia, and serotonin syndrome [75].

Rasagiline: Rasagiline is a second-generation MAO-B inhibitor. Like selegiline, rasagiline acts irreversibly and has been proven to be effective as a monotherapy for PD [71]. A double-blind randomized controlled trial of rasagiline on 56 PD patients demonstrated that rasagiline significantly improved PD symptoms when compared to placebo (p<0.05) with no significant difference in the occurrence of adverse effects [76]. Most critically, unlike selegiline, rasagiline appears to demonstrate disease-modifying effects. It is still debated and inconclusive. The ADAGIO study examined rasagiline’s disease-modifying effects in a large-scale, double-blind trial. The study involved 1,176 untreated PD patients randomly assigned to either an early-start group receiving rasagiline for 72 weeks or a delayed-start group that received a placebo for 36 weeks followed by rasagiline for the remaining 36 weeks. Results indicated that early-start treatment with rasagiline at a dose of 1 mg per day showed a significant superiority to placebo in the rate of UPDRS score change from weeks 12 to 36 (p = 0.01) and was superior to delayed-start treatment in the change from baseline to week 72 (p = 0.02), while also maintaining noninferiority in the rate of change between weeks 48 and 72 (p<0.001).

Thus, the early initiation of rasagiline treatment at a dose of 1 mg per day not only improves clinical symptoms of PD but also suggests a potential for slowing disease progression [77]. Later studies would confirm these findings, demonstrating that patients who started rasagiline earlier had less progression of symptoms than those who started the drug later and adding credence to a disease-modifying effect [78]. Rasagiline is fairly well-tolerated, with some adverse reactions being nausea, vomiting, orthostatic hypotension, somnolence, hallucinations, and dyskinesias. However, these adverse reactions are tolerable in most cases [79]. Furthermore, unlike selegiline, rasagiline metabolites do not have amphetamine-like side effects [80].

Safinamide: Safinamide, a selective and reversible MAO-B inhibitor, was the newest addition to this drug category in the US market in 2017, offering 95% bioavailability and available as an oral formulation in doses of 50 or 100 mg per day to reduce dyskinesia [81]. Its high selectivity and reversibility for MAO-B confer a significant advantage, as it reduces the risk of fatal hypertensive crises caused by the buildup of toxic amine metabolites, thereby eliminating dietary restrictions associated with amine-rich foods such as cheese and wine.

Studies using rat brain mitochondria have shown that safinamide exhibits 5,000 times greater selectivity for MAO-B over MAO-A, compared to the more modest selectivity ratios of 127 times for selegiline and 103 times for rasagiline [82]. Additionally, its reversibility minimizes the risk of potential drug interactions. Recent research has also highlighted safinamide’s ability to inhibit sodium and N-type calcium channels, which in turn suppresses potassium ion-mediated glutamate release and reduces NMDA receptor stimulation [83]. As a result, safinamide has demonstrated dual efficacy in reducing OFF time, increasing ON time, and improving non-motor symptoms such as pain, sleep disturbances, and mood disorders.

A 24-week, multicenter, phase 3 randomized, double-blind, placebo-controlled trial involving 669 patients with mid-to-late-stage PD on a stable LD regimen was one of the first large studies conducted. Participants treated with safinamide (50 mg/day or 100 mg/day) experienced significant improvements in ‘ON’ time compared to placebo, with mean increases of 1.3 hours in the safinamide groups versus 0.7 hours in the placebo group (50 mg/day, p = 0.022; 100 mg/day, p = 0.013). Additionally, reductions in complications of therapy were reflected in improved scores on the UPDRS Part IV [84].

The majority of participants continued into Study 018, a two-year extension study evaluating safinamide 100 mg/day. The primary endpoint was the change in Dyskinesia Rating Scale (DRS) total score during ON-time over 24 months, but no overall difference in dyskinesia was observed between safinamide and placebo groups, despite a decrease in mean DRS scores with safinamide and an almost unchanged score with placebo. However, ad hoc subgroup analysis revealed significant improvement in patients with moderate to severe dyskinesia at baseline (36% of participants), with safinamide reducing DRS scores compared to placebo (27% vs. 3%; p = 0.0317). Improvements in ON-time without troublesome dyskinesia, OFF-time, motor function, activities of daily living, depressive symptoms, and quality of life observed at six months were sustained throughout the two years [85,86]. Safinamide is associated with an increased risk of fractures and falls when used with anxiolytics or antihypertensives due to its hypotensive potential, psychosis when combined with amantadine, and neuropsychiatric adverse events with dopamine agonists. It also interacts with amantadine and SSRIs, raising the risk of serotonin syndrome [82]. The findings are summarized in Table 4.

**Table 4:** MAO-B inhibitors in the management Parkinson’s disease MAO-B: monoamine oxidase-B; UPDRS: Unified Parkinson's Disease Rating Scale; PD: Parkinson’s disease

Study; drug	Study population	Mechanism of action	Benefits (B) and risks (R)
Mizuno et al., 2017, Japan [72]; selegiline	N = 292	Irreversible MAO-B inhibitor	B: reduction of Parkinsonian symptoms. R: constipation, insomnia, hypertension, thirst, abdominal discomfort
Olanow et al., 2009, worldwide [77]; rasagiline	N = 1176	Irreversible MAO-B inhibitor	B: improvement in UPDRS score, possible disease-modifying effect. R: headache, back pain, depression, nasopharyngitis, anxiety, fatigue
Borgohain et al., 2014, worldwide [84]; safinamide	N = 669	MAO-B inhibitor and glutamate release modulator	B: improvement in “off” time, Increase in total “on” time, improvement of motor symptoms. R: dyskinesia, worsening PD, caratact, back pain, depression, headache, hypertension

Anticholinergics

Anticholinergic medications were among the earliest treatments for PD, specifically targeting motor symptoms such as tremors. These drugs, including trihexyphenidyl and benztropine, act by blocking muscarinic receptors, reducing the overactivity of cholinergic neurons that contribute to PD symptoms. However, their clinical utility has declined significantly following the introduction of LD and DAs, which offer superior efficacy with fewer side effects [87]. While anticholinergics can still be prescribed for PD tremors, their effectiveness is generally lower than that of LD. As such, they are typically reserved for younger patients without cognitive impairment, given their significant risk of neuropsychiatric and cognitive side effects [88]. The primary concern with anticholinergic medications in PD is their association with cognitive decline and dementia. Studies have shown that chronic exposure to these drugs increases the risk of dementia in both the general elderly population and individuals with PD, who are already at heightened risk for cognitive deterioration. Acute effects, such as delirium, have also been observed in PD patients receiving anticholinergics [87]. These are rarely used now except in specific younger patients.

The rationale behind using anticholinergic medications in PD stems from the intricate balance between dopaminergic and cholinergic neurotransmission in the basal ganglia. In PD, the degeneration of dopaminergic neurons in the substantia nigra leads to an imbalance, with increased cholinergic activity contributing to motor symptoms like tremor and rigidity. By inhibiting muscarinic receptors, anticholinergics help restore a more balanced interplay between these pathways. However, this approach is limited by the widespread distribution of cholinergic neurons throughout the brain, leading to off-target effects such as cognitive impairment and psychiatric disturbances [88]. Despite these risks, anticholinergic medications remain commonly prescribed for non-PD-related indications, such as urological, psychiatric, and cardiovascular conditions. A significant proportion of PD patients, between 31.5% and 46.3%, are on at least one anticholinergic medication, often without their primary indication being PD. The co-prescription of anticholinergic drugs with cholinesterase inhibitors, which are used to treat dementia, further complicates management, as these medications have opposing effects on cognitive function [89].

Trihexyphenidyl: Trihexyphenidyl is an anticholinergic agent commonly used to manage symptoms of PD, including tremors, stiffness, and muscle spasms. The medication was FDA-approved in 2003 for idiopathic, postencephalitic, and arteriosclerotic parkinsonism and is frequently used as an adjunct therapy alongside LD. The mechanism of action of trihexyphenidyl is not fully understood, but it has been suggested to interact with dopamine and M1 muscarinic receptors. Although generally well-tolerated, trihexyphenidyl can cause various dose-dependent adverse effects, including dry mouth, constipation, tachycardia, dizziness, confusion, and sedation. In elderly patients, the risk of cognitive impairment and delirium is significantly increased, making it potentially inappropriate for older adults [90]. Rare but severe adverse reactions have been reported, such as malignant hyperthermia. A case study described a 55-year-old PD patient with pneumonia who developed malignant hyperthermia and syndrome of inappropriate antidiuretic hormone secretion (SIADH) due to trihexyphenidyl. This highlights the need to consider drug-induced fever as a differential diagnosis in febrile patients on trihexyphenidyl therapy [91].

Emerging research suggests that trihexyphenidyl influences metabolism, neurobehavioral patterns, and gut microbiota. Chronic use has been associated with withdrawal symptoms, including heightened anxiety, depressive-like behaviors, increased cortisol levels, and oxidative stress. These effects are linked to gut dysbiosis, emphasizing the importance of considering microbiome interactions in long-term trihexyphenidyl therapy. Co-administration of Anacyclus pyrethrum has shown promise in mitigating withdrawal-related oxidative damage and restoring gut microbiota balance, suggesting potential therapeutic interventions for trihexyphenidyl withdrawal syndrome [92].

Benztropine: Benztropine is a synthetic muscarinic receptor antagonist classified as an anticholinergic drug, structurally related to diphenhydramine and atropine. It is FDA-approved as an adjunctive therapy for various forms of Parkinsonism, including idiopathic and postencephalitic Parkinsonism. Benztropine acts in the central nervous system (CNS) and smooth muscles by competing with acetylcholine at muscarinic receptors, reducing central cholinergic effects, which alleviates PD symptoms. It also demonstrates lower CNS stimulation than trihexyphenidyl, making it the preferred option for geriatric patients [93]. However, benztropine use is associated with notable adverse effects, including cognitive impairment, dry mouth, urinary retention, constipation, and blurred vision. Severe adverse effects include toxic megacolon, ocular hypertension, and paralytic ileus. Studies suggest that while benztropine is effective for drug-induced parkinsonism (DIP), it may exacerbate tardive dyskinesia (TD) and should be avoided in patients with comorbid TD. Alternative therapies such as amantadine may provide similar benefits with a lower risk of cognitive impairment [94].

Withdrawal from benztropine can also lead to withdrawal-emergent dyskinesia, as documented in a case report describing acute-onset dyskinesia following benztropine discontinuation [95]. In clinical practice, the use of benztropine requires careful consideration of patient-specific factors, including age, cognitive function, and comorbid conditions. While it remains an effective treatment for DIP and PD, its side effect profile necessitates cautious prescribing, particularly in elderly populations or individuals at risk of cognitive decline [93, 95]. The findings are summarized in Table 5.

**Table 5:** Anticholinergics in the management of Parkinson’s disease

Study; drug	Study population	Mechanism of action	Benefits (B) and risks (R)
Zhao et al., 2020, China [91]; trihexyphenidyl	N = 1	Muscarinic receptor antagonist	R: malignant hyperthermia, dry mouth, blurred vision, bradycardia, nausea, vomiting, urinary retention, constipation, erythema
Thalner and Agrawal, 2023, USA [95]; benztropine	N = 1	Muscarinic receptor antagonist with mild dopamine reuptake inhibition	R: withdrawal symptoms, dyskinesia, psychosis

N-Methyl-D-Aspartate Receptor Antagonists

PD, as a neurodegenerative disease, is marked by the accumulation of misfolded proteins, which lead to neuronal damage and cognitive decline. NMDA receptors, highly expressed in the basal ganglia, play a key role in excitatory synaptic transmission due to their calcium permeability and downstream signaling cascades. Activation requires ligand binding at both glutamate and glycine sites, while magnesium blockade at resting membrane potential ensures voltage-dependent activity [96]. Excessive stimulation of these receptors can lead to Ca²⁺ influx, triggering pathological changes and neuronal damage, contributing to excitotoxicity. In PD, dopamine depletion and LD treatment alter NMDA receptor subunit distribution, with increases in GluN2A, GluN2D, and GluN1 observed in PD models and patients [97, 8]. These findings suggest that NMDA receptor antagonists may serve as adjunct therapies to enhance the efficacy and tolerability of dopaminergic treatments and combinations with other antagonists. However, the widespread expression of NMDA receptors and their broad excitatory effects raise concerns that global inhibition could result in side effects such as ataxia, impaired learning, and psychosis [99].

Amantadine: A phase 3 trial was conducted to test the efficacy of the drug on alleviating dyskinesia and OFF time for PD patients and LD-induced dyskinesia. Several small studies have suggested that immediate-release (IR) amantadine helps reduce dyskinesia, but its effectiveness, especially in patients new to amantadine, remains unclear [100]. ADS-5102 is an extended-release (ER) formulation of amantadine, designed for once-daily administration at bedtime. This formulation provides a sustained pharmacokinetic profile, maintaining high drug levels throughout the day when dyskinesia is most problematic [101]. The efficacy of ADS-5102 was assessed in two randomized trials, EASE LID and EASE LID 3, which included 121 and 75 patients, respectively. In their respective studies, 63 and 37 patients received ADS-5102, while 58 and 38 received a placebo. The combined analysis included 100 patients treated with ADS-5102 and 96 patients receiving a placebo. Two additional participants were included in the safety population but did not contribute post-baseline Unified Dyskinesia Rating Scale (UDysRS) data.

In all subgroups measured, the ADS-5102 group showed a greater benefit than placebo, with the treatment effect independent of baseline dyskinesia severity, as evidenced by the overlap of 95% CIs for 12-week changes in UDysRS scores in different patient subgroups. The mean benefit was greatest in patients with more severe dyskinesia (baseline UDysRS total score ≥ 40 and MDS-UPDRS item 4.2 score 3-4). Significant improvements in daily PD clinical states, including a mean decrease in OFF time of approximately one hour per day by week 12 (p<0.0001), were observed in the ADS-5102 group, with complete resolution of OFF time and ON time with troublesome dyskinesia occurring more frequently compared to placebo.

The clinical relevance of these changes was further supported by the reduction in mean OFF time, which correlated with a reduction in ON time with troublesome dyskinesia. ADS-5102 was generally well tolerated, though visual hallucinations were reported more frequently in patients receiving the treatment, especially among older patients (≥65 years) or those with cognitive impairment, with dry mouth, constipation, and nausea also occurring due to its anticholinergic properties [102]. Amantadine (especially in extended-release formulation) is the only recognized, guideline-supported treatment for dyskinesia in PD. The findings are summarized in Table 6.

**Table 6:** NMDA receptor antagonists in the management of Parkinson’s disease NMDA: N-methyl-D-aspartate glutamate receptors

Study; drug	Study population	Mechanism of action	Benefits (B) and risks (R)
Elmer et al., 2018, USA [102]; amantadine	N = 196	NMDA antagonist	B: significant improvement from placebo for treatment of dyskinesia and “OFF” time. R: hallucinations, dizziness, dry mouth, peripheral edema, constipation, falls, orthostatic hypotension

Adjunctive Therapy

Adjunctive therapies are often necessary in the comprehensive management of PD, especially as patients experience motor fluctuations, psychosis, or cognitive decline that are not adequately addressed by LD alone. These treatments target non-dopaminergic systems or provide complementary mechanisms to enhance symptom control without exacerbating motor complications. Clazapine is highly effective for refractory PD psychosis but requires regular neutrophil and white blood cell count monitoring carries risk of sedation, orthostatic hypotension, and myocarditis. This section highlights three key adjunctive agents: pimavanserin, a selective 5-HT₂A inverse agonist approved for PD psychosis (PDP) [103-105]; rivastigmine, a cholinesterase inhibitor used to treat cognitive impairment and gait instability in PD dementia [106-108]; and istradefylline, an adenosine A2A receptor antagonist used to reduce OFF episodes in patients with motor fluctuations [109]. Together, these agents reflect the growing importance of individualized, multi-targeted strategies in PD management.

Pimavanserin: Pimavanserin, a 5-HT₂A inverse agonist used to treat hallucinations and delusions associated with PDP, is the only FDA-approved atypical antipsychotic for PDP [103]. It works to combat psychosis linked to anticholinergic medications and DA-induced hallucinations. Pimavanserin differs from typical antipsychotics because it doesn't affect dopamine levels, specifically at the D2 receptor. It's the first drug to show antipsychotic effects without blocking dopamine D2, which helps manage psychotic symptoms in Parkinson's without worsening its motor symptoms [104]. In a six-week randomized, double-blind, placebo-controlled trial, pimavanserin significantly reduced SAPS-PD (Scale for the Assessment of Positive Symptoms in Parkinson’s Disease) scores compared to placebo (-5.79 vs -2.73; 95% CI -4.91 to -1.20; p = 0.001), demonstrating its efficacy in alleviating psychotic symptoms in PD without worsening motor function [105]. Unlike other drugs such as quetiapine or clozapine used for Parkinson's psychosis, pimavanserin doesn't cause drowsiness [104].

Rivastigmine: Rivastigmine is a cholinesterase inhibitor used for dementia in individuals with mild to moderate PD [106]. It works by preventing the breakdown of acetylcholine in the brain, which in turn boosts its levels and improves cholinergic transmission. Rivastigmine reversibly binds and inhibits both acetylcholinesterase and butyrylcholinesterase, helping counteract the increased levels of these enzymes in aging, Alzheimer’s, and PD [107]. Besides treating dementia, rivastigmine is also noted to improve gait stability. In a 32-week trial, PD patients receiving rivastigmine had reduced step time variability during normal walking (ratio of geometric means 0.72, 95% CI 0.58-0.88; p=0.002) and simple dual tasks (0.79, 95% CI 0.62-0.99; p=0.045), though side effects like nausea and vomiting were more frequent in the treatment group (p<0.0001) [108]. The adverse effects of this medication are mainly gastrointestinal, with less common ones related to extrapyramidal symptoms, sleep disturbances, muscle cramps, and weakness. Some recent studies have found that, compared to oral administration, the transdermal path for rivastigmine provides consistent, controlled delivery over 24 hours with fewer side effects [106].

Istradefylline: Istradefylline offers a novel non-dopaminergic adjunct therapy to LD for managing “OFF” episodes in individuals with moderate to severe PD [109]. As a xanthine derivative, istradefylline acts within the basal ganglia by selectively targeting adenosine A2A receptors, which influence output pathways involving glutamate and GABA, both of which are largely regulated independently of dopamine. These receptors, found predominantly on dopamine D2-containing neurons, mainly affect the indirect pathway and serve as key targets for adenosine antagonists to reverse motor dysfunction [110] and modulate striatal synaptic plasticity [109]. A meta-analysis of six studies (n = 1175 istradefylline; n = 643 placebo) confirmed that 40 mg/day consistently reduced OFF time (SMD = -0.28, 95% CI = -0.44 to -0.12, p = 0.0005), while the effect of 20 mg/day was less consistent (SMD = -0.23, 95% CI = -0.40 to -0.06, p = 0.009) and lost when certain studies were excluded.

Istradefylline also improved UPDRS Part III scores during the ON phase at both doses, with a stronger effect at 40 mg/day (SMD = -0.24, 95% CI = -0.37 to -0.11, p = 0.0002) than at 20 mg/day (SMD = -0.15, 95% CI = -0.27 to -0.02, p = 0.02). Although istradefylline was associated with a significantly higher risk of dyskinesia compared to placebo (RR = 1.72, 95% CI = 1.26 to 2.34, p = 0.0007), no significant differences were observed for mild adverse reactions such as tremor, nausea, constipation, hallucinations, insomnia, somnolence, or accidents. Collectively, these findings support the efficacy of istradefylline at doses of 20 and 40 mg/day in reducing OFF time and improving motor function in PD patients with motor fluctuations [111].

Istradefylline is currently approved for treating OFF episodes in PD based on clinical trials conducted in Japan and the U.S. However, as the first adenosine A2A receptor antagonist used in PD, its full therapeutic potential remains unclear and requires further investigation in real-world settings. Emerging data from Japan indicate that istradefylline may also improve motor and non-motor symptoms, including gait impairments, neuropsychiatric disturbances, sleep disruptions, and urinary dysfunction [112-115]. Although these preliminary findings are encouraging, larger, well-controlled phase 4 studies are necessary to validate their effectiveness in these areas. The findings are summarized in Table 7.

**Table 7:** Adjunctive therapy in the management of Parkinson’s disease

Study; drug	Study population	Mechanism of action	Benefits (B) and risks (R)
Cummings et al., 2014, USA/Canada [105]; pimavanserin	N = 314	5-HT₂A inverse agonist	B: alleviating psychotic symptoms in Parkinson’s disease without worsening motor function, no drug-associated drowsiness. R: urinary tract infection, fall, prolonged QT, confusion, hallucinations
Henderson et al., 2016, UK [108]; rivastigmine	N = 130	Cholinesterase inhibitor	B: reduced step time variability during normal walking and simple dual tasks. R: nausea and vomiting
Sako et al., 2017, Japan [111]; istradefylline	N = 1175	Adenosine A2A receptor antagonist	B: decreased off time and improved motor symptoms. R: higher risk of dyskinesia

Novel treatments

Lysosomal enhancer-associated* *dysfunction plays a central role in the pathogenesis of PD by impairing the degradation of alpha-synuclein (α-Syn), leading to the accumulation of toxic aggregates that exacerbate neuronal damage [116]. Normally, lysosomes are responsible for degrading α-Syn and maintaining cellular homeostasis, but when this process is disrupted, it contributes significantly to disease progression. Mutations or deficiencies in glucocerebrosidase (GBA) associated with PD trigger a positive feedback loop in which impaired lysosomal function promotes α-synuclein accumulation. This, in turn, further disrupts GBA activity by hindering its trafficking from the endoplasmic reticulum to the Golgi to lysosomes, ultimately contributing to neurodegeneration [117].

Similarly, mutations in the ATP13A2 gene associated with PD result in widespread lysosomal impairment, characterized by membrane instability, impaired acidification, reduced processing of lysosomal enzymes, and diminished degradation of substrates and autophagosomes [118]. Together, these mechanisms converge to promote α-Syn accumulation, exacerbate lysosomal dysfunction, and thus contribute to cell death in PD. Therapies such as ambroxol, a small molecule with lysosomal-enhancing properties, and buntanetap tartrate, a compound that can reduce neurotoxic protein levels, are some of the novel therapies currently under investigation for their potential to restore cellular balance and slow the progression of PD.

Ambroxol

Mutations in the GBA gene are a significant genetic risk factor for PD and are found in approximately 5-15% of patients. These mutations result in decreased lysosomal function, impairing the processing of α-Syn and leading to lipid accumulation and subsequent neurodegeneration. Additionally, GBA mutations are linked to reduced glucosyl ceramidase activity and increased oxidative stress [119]. Given its central role in the pathological development of PD, GBA deficiency has become a target for therapeutic intervention. A recent study investigated fibroblast skin cell cultures from patients with Gaucher’s disease and PD, both of which exhibit reduced GBA activity. Treatment with ambroxol hydrochloride significantly increased glucosylceramidase activity in fibroblasts from healthy controls, Gaucher disease patients, and heterozygous glucocerebrosidase mutation carriers with PD. This enzymatic enhancement was accompanied by a 50% reduction in dihydroethidium oxidation rate (p<0.05) across all groups. These findings support ambroxol’s potential to improve lysosomal function and mitigate oxidative stress in these populations [120].

Another single-center, open-label, noncontrolled clinical trial evaluated escalating-dose oral ambroxol therapy (up to 1.26 g/day, administered as 420 mg three times daily) in patients with moderately severe PD. Between baseline and 186 days, total CSF α-synuclein concentration increased by 50 (17) pg/mL (13%; 95% CI, 14-87; p = .01), and CSF GCase protein levels increased by 88 (22) pmol/L (35%; 95% CI, 40-137; p = .002), suggesting enhanced enzyme delivery and lysosomal engagement in the CNS. Notably, CSF tau (p = .36) and glucosylceramide (P = .16) levels remained unchanged, supporting the absence of accelerated neurodegeneration. Blood leucocyte GCase activity showed no significant change (p = .48), while serum tau levels decreased by 0.20 (0.08) pg/mL (95% CI, -0.37 to -0.05; p = .01), and serum α-Syn concentrations exhibited wide variability (p = .46).

Motor symptoms, measured by the MDS-UPDRS, improved with a mean decrease of 8.7 (11.8) points (95% CI, -15.3 to -2.2; p = .01) between baseline and 186 days, largely driven by a 6.8 (7.1) point decrease in part 3 motor scores (95% CI, -10.4 to -3.1; p = .001), followed by a rebound between 186 and 279 days. Cognitive performance showed a small improvement in Montreal Cognitive Assessment (MoCA) scores (mean increase of 1.7 [1.3] points), while non-motor symptoms worsened, reflected by an increase in Non-Motor Symptoms Scale (NMSS) scores of 11.5 (18.5) points (95% CI, 2.4-20.8; p = .02).

Adverse events were reviewed and categorized by the investigators according to their presumed relationship to ambroxol. Events deemed “probably related” included nausea, vomiting, a burning sensation after swallowing, and loose stool. “Definitely related” events were acid reflux, nausea, and a transient skin condition on the chest, back, and arms [121]. A phase 2 clinical trial is currently underway to evaluate the efficacy of ambroxol in PD. This phase 2 multicenter, double-blind, placebo-controlled trial randomized patients with GBA-PD to receive either oral ambroxol 1.2 g/day or placebo for 52 weeks. This trial, which began in January 2023, is expected to conclude in December 2024 and may offer a new therapeutic option for patients with GBA-associated PD [122].

Buntanetap Tartrate

It is believed that buntanetap inhibits the synthesis of α-synuclein. By reducing its production, the drug may slow the accumulation of α-synuclein and mitigate its pathological effects. To study this, a double-blind, placebo-controlled, combined phase 1/2 trial evaluating the effects of buntanetap on Alzheimer’s disease and PD was conducted across 13 U.S. sites. The trial included 54 patients with early-stage PD who received varying doses of buntanetap or placebo. Statistical comparisons were made between post-treatment and baseline values for both the placebo and buntanetap groups, as well as between buntanetap and placebo post-treatment outcomes.

Over the 25 ± 2-day treatment period, buntanetap, particularly at a 10 mg once-daily dose, dose-significantly improved patients’ MDS-UPDRS Part III motor scores. Both the 10 mg and 20 mg once-daily groups also showed significant improvements in total MDS-UPDRS scores compared with baseline. Additionally, the 5 mg once-daily dose significantly improved patients’ Wechsler Adult Intelligence Scale (WAIS) coding scores compared to both baseline and placebo (P < 0.05; P < 0.01). Biomarker analyses further suggested a trend toward reduced levels of neurotoxic proteins and improved axonal integrity, alongside statistically significant improvements in cognitive function [123]. Most adverse events were related to study procedures (lumbar puncture), with no clinically significant findings in vital signs or physical exams. A single Grade 1 QT prolongation in a patient receiving buntanetap resolved and was deemed non-clinically significant. Reported treatment-emergent adverse events were headache, erythema, movement disorder, and muscle spasms [123]. The findings are summarized in Table 8.

**Table 8:** Lysosomal enhancers in the management of Parkinson’s disease GCase: β-glucocerebrosidase; MDS-UPDRS: The International Parkinson and Movement Disorder Society sponsored revision of the Unified Parkinson's Disease Rating Scale

Study; drug	Study population	Mechanism of action	Benefits (B) and risks (R)
Mullin et al., 2020, United Kingdom [121]; ambroxol	N = 17	GCase enzyme enhancer	B: mean total MDS-UPDRS score decreased (ie, improved) by 8.7 (11.8) points. R: acid reflux, nausea, and a transitory skin condition on the chest, back, and arms
Fang et al., 2023, USA [123]; buntanetap	N = 54	Neurotoxic protein suppressor	B: The 10 mg and 20 mg once-daily group showed significant improvement compared to baseline. R: Headache, erythema, movement disorder, and muscle spasms

*Neuroprotective Agents* *Glucagon-Like Peptide-1 Receptor Agonist (GLP-1RA)*

Although PD is predominantly known as a neurodegenerative disorder, recent research suggests a link between PD and type 2 diabetes mellitus (T2DM), particularly through the gut-brain axis. GLP-1 is a multifunctional peptide produced in the intestines and brainstem, influencing neurogenesis, neurodegeneration, energy homeostasis, and other processes. It binds to GLP-1 receptors, expressed in pancreatic cells and key brain regions like the substantia nigra, striatum, and cortex, where it modulates neuronal, microglial, and astrocytic functions. GLP-1’s ability to cross the blood-brain barrier, improve endothelial function, suppress inflammation, and protect neurons makes it a promising therapeutic target for PD [124, 125]. GLP-1RA, originally developed for managing diabetes mellitus, has demonstrated significant neuroprotective properties through mechanisms such as enhancing mitochondrial biogenesis, reducing oxidative stress, and modulating neuroinflammatory responses [126,127]. These agonists, including exenatide and liraglutide, upregulate peroxisome proliferator-activated receptor gamma coactivator 1-alpha (PGC-1α), restoring mitochondrial function and improving neuronal survival while decreasing pro-inflammatory cytokines and promoting an anti-inflammatory environment [128].

Exenatide: Exenatide is one of the first GLP-1 receptor agonists to be evaluated in clinical trials for PD. Based on its established safety profile in type 2 diabetes [129] and promising neuroprotective effects in preclinical studies [130], it has become a leading candidate in efforts to repurpose metabolic therapies for PD. In a single-center, randomized, double-blind, placebo-controlled trial, patients with moderate PD received their standard medication alongside weekly subcutaneous injections of either 2 mg exenatide or placebo for 48 weeks, followed by a 12-week washout period. 62 patients were enrolled in the study, with 32 receiving exenatide and 30 receiving placebos. The primary analysis included 31 and 29 patients from each group, respectively. Injection site reactions and gastrointestinal symptoms were common adverse events in both groups. Six serious adverse events occurred in the exenatide group and two in the placebo group, with none attributed to the study interventions.

After 60 weeks, off-medication scores on MDS-UPDRS Part III improved by 1.0 point (95% CI: -2.6 to 0.7) in the exenatide group, whereas the placebo group experienced a decline of 2.1 points (95% CI: -0.6 to 4.8). This yielded an adjusted mean difference of -3.5 points, which was statistically significant (p = 0.0318). This finding suggests that exenatide not only improves symptoms during treatment but also provides residual benefits after discontinuation [131]. Overall, exenatide has demonstrated positive effects on motor scores in PD patients, further supporting its potential therapeutic value. Additional research also suggests that diabetic patients treated with exenatide may have a reduced risk of developing PD [132]. Building on these promising results, a phase 3 trial is currently underway, involving 200 patients over two years. This trial aims to evaluate the long-term efficacy of exenatide to achieve a greater than 2.5-point improvement on MDS-UPDRS Part III. The trial is expected to conclude by the end of 2024 [127].

Liraglutide: Liraglutide, a recombinant GLP-1 analog, is characterized by its delayed absorption and a prolonged plasma half-life of over 13 hours, owing to its binding with albumin. This feature sets it apart from short-acting GLP-1R agonists, as liraglutide tends to cause fewer side effects while yielding more significant reductions in glycated hemoglobin and fasting blood glucose levels [124]. Although only one phase 2 clinical trial has assessed liraglutide's effects in PD, the results are promising. In a single-center, randomized, double blind, placebo-controlled trial of 63 patients, 42 were randomized to receive liraglutide, while 21 were given a placebo. After 54 weeks, the liraglutide group saw a 6.6-point improvement in NMSS scores, compared to a 6.5-point worsening in the placebo group (adjusted mean difference: 13.1, p=0.07). While there were no significant changes in MDS-UPDRS Part III or Mattis Dementia Rating Scale, Second Edition (MDRS-2) scores, significant improvements were observed in MDS-UPDRS Part II scores of the liraglutide group (−4.1 points, p=0.001). Common adverse events included injection site reactions and gastrointestinal symptoms [133]. The findings are summarized in Table 9.

**Table 9:** GLP-1 receptor agonists in the management of Parkinson’s disease MDS-UPDRS: The International Parkinson and Movement Disorder Society sponsored revision of the Unified Parkinson's Disease Rating Scale; GLP-1: glucagon-like peptide-1

Study; drug	Study population	Mechanism of action	Benefits (B) and risks (R)
Athauda et al., 2017, United Kingdom [131]; exenatide	N = 60	GLP-1RA	B: improvement in off-medication scores in MDS-UPDRS Part III. R: injection site reaction, gastrointestinal symptoms, weight loss, nausea
Malatt et al., 2022, United States [133]; liraglutide	N = 55	GLP-1RA	B: MDS-UPDRS Part II improvement. R: injection site reaction, gastrointestinal symptoms

Gastrointestinal Modulating Agents

Gastrointestinal dysfunction is a prevalent, yet under-recognized component of PD. Emerging evidence indicates that PD may potentially begin in the gut, with early accumulation of α-Syn in the enteric nervous system contributing to dysmotility and other gastrointestinal disturbances [134]. Symptoms such as dysphagia, gastroparesis, and chronic constipation are not only common in PD but also serve to significantly degrade the patients’ quality of life, leading to complications like malnutrition and aspiration pneumonia. Diagnostic advances have enabled more refined evaluations of these impairments, while management remains challenging due to the limited availability of effective treatments [135]. Probiotics, such as Lactobacillus acidophilus, are promising avenues currently being investigated to target the gut-brain axis via modulation of the gut microbiome.

Lactobacillus acidophilus: Lactobacillus acidophilus is a well-known probiotic strain widely recognized for its beneficial effect on gut health. It has been studied extensively for its ability to maintain gut microbial balance, support digestion, and reduce inflammation. Furthermore, recent research suggests that Lactobacillus acidophilus may also have potential therapeutic benefits for neurodegenerative conditions such as PD. In a phase 2 Trial, a randomized, double-blind, placebo-controlled clinical trial involving 60 people with PD. Participants were divided into two groups, with one group receiving a probiotic dose of 8 × 10^9 ^colony-forming units (CFU)/day and the other receiving a placebo, over 12 weeks. MDS- UPDRS was used to record changes in motor function. Probiotic supplementation led to a significant improvement in MDS-UPDRS scores, indicating enhanced motor function. It also resulted in decreased levels of high-sensitivity C-reactive protein and malondialdehyde, which are markers of inflammation and oxidative stress, respectively [136].

Another phase 2 trial, a randomized, double-blind, placebo-controlled clinical trial, involved 72 people with PD. Participants were block-randomized to receive either multistrain probiotic capsules (34 patients) or a placebo (38 patients) for a duration of four weeks. The main measure was the change in the average number of spontaneous bowel movements (SBM) per week, comparing the last two weeks of the intervention with the two-week preintervention phase, documented via a daily stool diary. The study concludes that multistrain probiotics are effective for alleviating constipation in PD. However, it highlights the need for further research to explore the long-term efficacy and safety of probiotics in this patient population, as well as to understand the mechanisms by which they exert their effects [137]. Multiple ongoing clinical trials are investigating the efficacy of Lactobacillus Acidophilus. This includes a phase 3 trial assessing the long-term efficacy and safety of this intervention, which is currently recruiting participants with completion expected in June 2025 [138], as well as a phase 4 trial assessing the role and mechanism of Bifidobacterium triple viable capsules, with expected completion in December 2023 [139]. The findings are summarized in Table 10.

**Table 10:** Gastrointestinal modulating agents in the management of Parkinson’s disease MDS-UPDRS: The International Parkinson and Movement Disorder Society sponsored revision of the Unified Parkinson's Disease Rating Scale; CRP: C-reactive protein; PD: Parkinson’s disease

Study; agent	Study population	Mechanism of action	Benefits (B) and risks (R)
Tamtaji et al., 2018, Iran [136]; Lactobacillus acidophilus	N = 60	Probiotic gut microbiota modulator	B: improved MDS-UPDRS score, reduction of high-sensitivity CRP, and enhanced glutathione levels. Statistically significant reduction in insulin levels
Tan et al., 2021, Malaysia [137]; Lactobacillus acidophilus	N = 72	Probiotic gut microbiota modulator	B: alleviation of constipation in PD

Novel Dopaminergic Therapies

PD is well known and characterized by motor symptoms, which are driven by the progressive loss of dopaminergic neurons. While current therapies focus on replenishing or modulating dopamine to alleviate symptoms, new therapies, including novel drugs and delivery systems, are being explored to enhance motor control while minimizing side effects, offering an improved quality of life for PD patients.

Tavapadon: Tavapadon, also known by the developmental name PF-06649751, is an oral, highly selective partial agonist for D1/D5 receptors. Unlike full agonists, which cause strong, repeated stimulation of D2/D3 dopamine receptors and have historically caused cardiovascular issues and had poor pharmacokinetics, tavapadon is designed to offer motor control benefits without the severe side effects [140]. Early-phase studies have provided promising data on tavapadon’s safety and efficacy profile. Phase 1 clinical trials demonstrated that tavapadon was well tolerated in subjects with PD, with dose-dependent reductions in MDS-UPDRS Part III scores. Peak plasma concentrations were reached within one to four hours, and the drug demonstrated sustained pharmacodynamic effects, maintaining motor improvements up to 12 hours post-dose [141].

A phase 2 trial further investigated tavapadon’s efficacy in early-stage PD over 15 weeks. Though this study was terminated early, it was not due to safety concerns or poor results. It was linked to another trial testing Tavapadon in patients with more advanced PD. When that trial’s early analysis showed it was unlikely to reach its stricter efficacy goal, it was shut down, leading to the early termination of this trial as well. However, the 57 patients already enrolled were allowed to continue and finish the study. In the end, the study found that patients receiving tavapadon showed a statistically significant improvement in MDS-UPDRS Part III scores compared to placebo, with an average reduction of 9 points versus 4.3 in the placebo (p = 0.0407) [142].

Tavapadon is also currently undergoing a series of clinical trials. The TEMPO-1 trial assessed tavapadon at two fixed doses (5 mg and 15 mg, administered once daily) in adults diagnosed with early-stage PD. These developments are part of a comprehensive clinical program investigating tavapadon’s efficacy, safety, and tolerability across a diverse PD population. This program includes further phase 3 trials (TEMPO-2 and TEMPO-3) and an open-label extension study (TEMPO-4), aiming to substantiate the drug’s potential benefits over extended periods. Currently, phase 3 trials, such as the TEMPO-4 study, are evaluating its long-term efficacy in PD patients. This study is currently enrolling by invitation and is expected to be completed in January 2026 [143].

ND0612: ND0612 is a subcutaneous infusion system for continuous LD/CD delivery, which aims to prevent fluctuations in plasma levels, a limitation of oral LD. It is a drug-device combination consisting of a sterile solution of LD/CD delivered via a dedicated subcutaneous pump [144]. Previous phase 2 studies have found that ND0612 provides a well-tolerated and effective method to deliver LD/CD, improving motor control in PD patients. During one study, patients continued their standard of care (SoC) oral LD/CD and were randomized 2:1 to receive either ND0612 (daily dose of 270/63 mg LD/CD) or placebo infusion alongside SoC for 14 days [145]. This and other studies concluded that patients treated with ND0612 avoided deep troughs in LD plasma levels and had lower fluctuation indices compared to those on placebo [146].

Efficacy results further support its benefits, with the 24-hour ND0612 infusion group showing a significant reduction in OFF time (-2.8 hours [-4.6, -0.9]; p = 0.004) at Day 28. Notably, 63% of these patients achieved a ≥50% reduction in OFF time, and 42% experienced a complete reduction to zero hours. Additionally, the proportion of patients achieving full ON by 9:00 AM increased from 36.8% at Day one to 81.3% at Day 28, demonstrating improved morning symptom control. However, adverse events were frequent, with infusion site reactions (e.g., nodules, bruising, erythema) being the most common, though most were mild-to-moderate in severity [144,147]. Phase 3 trials, such as the BouNDless study, are ongoing to evaluate the long-term efficacy of ND0612. The study remains active, with expected completion in February 2027.

Apomorphine: Apomorphine is a short-acting D1- and D2-like receptor agonist, noted for its efficacy comparable to LD but with quicker onset and shorter duration of effect. Apomorphine is a well-established dopamine agonist used for the treatment of OFF episodes in PD. It is available in various formulations, including subcutaneous apomorphine infusion (SC-APO) and, most recently, sublingual film (SL-APO), which have been shown to provide rapid relief from motor fluctuations.

SC-APO has been employed for over 30 years as a device-aided therapy to maintain an ON state while minimizing dyskinesias. It is often indicated when oral medications fail to provide adequate symptom control and is preferred over more invasive interventions like deep brain stimulation (DBS) or LD/CD intestinal gel [148]. The TOLEDO study confirmed the long-term efficacy of SC-APO, showing sustained reductions in daily OFF time and improvements in ON time without troublesome dyskinesia for up to 64 weeks. Although generally well-tolerated, common adverse events include infusion site reactions, somnolence, and nausea, with a small percentage of patients discontinuing due to adverse effects [149].

SL-APO, a more recent formulation, has been evaluated for its convenience and patient preference. A randomized crossover study comparing SL-APO and SC-APO found no significant difference in motor improvement (MDS-UPDRS Part III score) but reported greater patient preference for SL-APO due to ease of administration and satisfaction [150]. Additionally, studies suggest that higher doses of SL-APO may provide enhanced motor benefits while remaining tolerable [144]. The necessity of prophylactic antiemetics during SL-APO initiation remains debated, as nausea and vomiting were uncommon in many patients, particularly those with prior DA use [151]. Despite its efficacy, apomorphine infusion remains underutilized, possibly due to misconceptions about its complexity and perceived status as a last-resort therapy. Clinical guidelines emphasize the need for early consideration of apomorphine infusion in treatment plans, supported by data demonstrating its significant impact on quality of life and reduction in oral medication dependence [148,149].

Foscarbidopa/foslevodopa: Foscarbidopa with foslevodopa represents an innovative subcutaneous delivery system for LD/CD, offering a 20:1 ratio and achieving faster steady-state plasma levels through the administration of an initial loading dose. This development aims to address the limitations of traditional oral therapies in managing PD, particularly in advanced stages where fluctuating dopamine levels lead to complications such as dyskinesias and "OFF" periods [152].

LD is the gold standard treatment for PD, functioning as a dopamine precursor that requires conversion in the central nervous system (CNS). To prevent premature peripheral decarboxylation of LD, which necessitates higher dosing and increases the risk of side effects, it is co-administered with CD, a peripheral decarboxylase inhibitor. Despite the efficacy of oral LD/CD in early PD, its effectiveness diminishes as the disease progresses, leading to significant motor fluctuations. The LD/CD intestinal gel (Duodopa) was developed to provide more stable dopamine levels but requires surgical implantation, underscoring the need for a less invasive, yet equally effective treatment [152]. A phase 1 study focused on assessing the solubility and stability of foslevodopa and foscarbidopa while maintaining consistent LD and CD plasma levels via subcutaneous infusion. In Groups 1 and 2, healthy volunteers achieved steady-state levels within 12-16 hours without a loading dose, showing higher CD bioavailability than oral administration, with an optimal 20:1 dosing ratio. Group 3, using a loading dose, reached steady-state levels within two hours, keeping a highly stable LD profile over 72 hours. This stability surpasses oral LD/CD and LD/CD intestinal gel, indicating better potential symptom control [152].

Another phase 3 trial further validated the efficacy of foslevodopa and foscarbidopa in PD patients. The study demonstrated significant improvements in "on time" without troublesome dyskinesia, with an increase of 2.72 hours compared to 0.97 hours with standard oral LD/CD (95% CI 0.46 to 3.05; p=0.0083). Additionally, "off time" was significantly reduced (95% CI -3.03 to -0.54; p=0.0054) by 2.75 hours compared to 0.96 hours with oral LD/CD [153]. ABBV-951 offers a promising new approach to PD management, combining the convenience of a subcutaneous delivery system with the ability to achieve stable and personalized LD plasma levels. Phase 1 and phase 3 studies underscore its potential to provide superior symptom control with reduced motor fluctuations, paving the way for improved quality of life in PD patients. Further research will continue to elucidate its full clinical benefits in this patient population.

Combination of extended-release 0.6 mg pramipexole and 0.75 mg rasagiline: Currently in phase 3 development for early PD, it is a fixed low-dose combination of extended-release 0.6 mg pramipexole and 0.75 mg rasagiline [154]. Pramipexole, a dopamine agonist, targets both pre- and post-synaptic dopamine receptors [155], offering over 90% oral bioavailability and is well-absorbed with minimal first-pass metabolism. However, it can cause side effects like daytime sleepiness and impulse control disorders, particularly at higher doses [154]. In contrast, rasagiline is a selective irreversible MAO-B inhibitor that prevents dopamine breakdown by binding permanently to the enzyme's flavin adenine dinucleotide (FAD) component [155]. Rasagiline has a lower bioavailability of about 36% and is extensively protein-bound, with fewer side effects compared to pramipexole, often similar to placebo [154].

The goal of P2B001 is to combine these two mechanisms to provide enhanced efficacy with fewer dopaminergic motor complications. In a 12-week, double-blind trial with 544 PD patients, P2B001 demonstrated great efficacy over its individual components. The mean difference in UPDRS II + III scores was −2.66 (95% CI, −4.33 to −1.00) versus pramipexole-ER 0.6 mg (P = 0.0018) and −3.30 (95% CI, −4.96 to −1.63) versus rasagiline-ER 0.75 mg (P < 0.0001). Additionally, P2B001 showed fewer side effects, with a significant reduction in daytime sleepiness compared to pramipexole ER, as measured by the Epworth Sleepiness Scale [154]. The findings are summarized in Table 11.

**Table 11:** Novel dopaminergic agents in the management of Parkinson’s disease MDS-UPDRS: The International Parkinson and Movement Disorder Society sponsored revision of the Unified Parkinson's Disease Rating Scale; LD/CD: levodopa/carbidopa; CGI-C: Clinical Global Impression of Change; UPDRS: Unified Parkinson's Disease Rating Scale; PDQ-39: Parkinson's Disease Questionnaire; DA: dopamine; MAO-B: monoamine oxidase-B

Study; drug	Study population	Mechanism of action	Benefits (B) and risks (R)
Riesenberg et al., 2020, USA [142]; Tavapadon	N = 57	Highly selective partial D1/D5 receptor agonist	B: improvement in MDS-UPDRS Part III score with reduced side effects compared to traditional D2/D3 agonists. R: nausea, headache, dry mouth, somnolence, tremor
Olanow et al., 2021 [144]; ND0612	N = 38	Continuous subcutaneous infusion of LD/CD via a dedicated pump	B: reduced OFF time, increased “ON” time, reduction in moderate-severe dyskinesia, improvement in CGI-C, UPDRS Part II, Part III, and PDQ-39 scores. R: infusion site reactions: nodules, bruising, erythema
Katzenschlager et al., 2021 [149]; apomorphine	N=84	Short-acting D1- and D2-like receptor agonist	B: rapid relief of OFF episodes, reduction in daily OFF time, increase in ON time without troublesome dyskinesia. R: infusion site reactions (SC), nausea, somnolence, potential impulse control disorders, rare: autoimmune hemolytic anemia
Soileau et al., 2023, Australia and USA [153]; ABBV-951 (foscarbidopa/foslevodopa)	N = 174	Continuous subcutaneous delivery of a 20:1 ratio of foslevodopa/ foscarbidopa	B: stable plasma LD levels, improved ON time without troublesome dyskinesia, and significantly reduced OFF time. R: infusion site reactions (erythema, pain, cellulitis, edema), typical dopaminergic effects (e.g., nausea, dyskinesia)
Hauser et al., 2022, USA [154]; P2B001	N = 544	Low-dose fixed combination of extended-release pramipexole (DA agonist) and rasagiline (MAO-B inhibitor)	B: improved UPDRS Part II + III scores, fewer side effects like somnolence, orthostatic hypotension, and neuropsychiatric effects vs. higher DA-agonist doses. R: nausea, fatigue, somnolence, dizziness, insomnia, headache

Affective Disorder Therapies

Nortriptyline/escitalopram: Depression is intricately linked to PD, with approximately 30-35% of individuals with PD experiencing clinically significant depressive symptoms [156]. However, there is a lack of robust evidence from concrete studies regarding the efficacy of SSRIs and TCAs in treating depression in this population, and no definitive conclusions have been drawn about their impact on persistent versus "off-time" affective disorders [157].

Selective serotonin reuptake inhibitors (SSRIs) such as escitalopram are widely accepted as the first-line treatment for depression. However, the use of SSRIs in PD is conservative due to concerns of worsening Parkinsonism [158]. SSRIs can also cause side effects such as fatigue and postural hypotension, further increasing the risk of falls, which is already elevated in patients with PD. Furthermore, SSRIs have been linked rarely to serotonin syndrome. In contrast, tricyclic antidepressants (TCAs) offer similar efficacy to SSRIs and have the advantage of anticholinergic properties that may alleviate tremors and insomnia in PD [157]. Among TCAs, nortriptyline has shown potential neuroprotective effects by inhibiting alpha-synuclein aggregation and neurotoxicity in preclinical studies [159]. However, TCAs are generally recommended as second-line treatments due to their higher risk of adverse effects, including orthostatic hypotension, dry mouth, constipation, urinary retention, memory impairment, hallucinations, and confusion [157].

Antidepressants Trial in Parkinson’s Disease (ADepT-PD) is an ongoing phase 3 randomized, double-blind, controlled pilot study aimed to assess the efficacy and cost-effectiveness of SSRI escitalopram compared to TCA nortriptyline in alleviating depressive symptoms in PD over eight weeks. There is a secondary aim to also evaluate nortriptyline's role in curbing the progression of Parkinson’s compared to a placebo group. Results from this study are pending, with final data expected to further refine our understanding of antidepressant therapy and its potential disease-modifying effects in PD. The findings are summarized in Table 12.

**Table 12:** Medications for the management of Parkinson’s disease with affective disorders PD: Parkinson’s disease

Study; drug	Study population	Mechanism of action	Benefits (B) and risks (R)
Schrag et al., 2022 UK [157]; nortriptyline	N = 408	Tricyclic antidepressant	B: anticholinergic properties that may alleviate tremor and insomnia, neuroprotective effects by inhibiting alpha-synuclein aggregation and neurotoxicity. R: orthostatic hypotension, dry mouth, constipation, urinary retention, memory impairment, hallucinations, and confusion
Schrag et al., 2022 UK [157]; escitalopram	N = 408	Selective serotonin reuptake inhibitors	B: treat PD depression. R: fatigue, postural hypotension, and an increased risk of falls, serotonin syndrome

Gene Modification Therapy

Approximately 20 causative genes are associated with familial PD, while over 200 genes have been implicated in sporadic PD. These genetic influences span both autosomal dominant and recessive inheritance patterns. The first autosomal dominant gene linked to PD was SNCA (PARK1/PARK4), which encodes alpha-synuclein. Point mutations in SNCA are believed to disrupt protein folding and promote aggregation [160]. Another key gene, Leucine-rich repeat kinase 2 (LRRK2), encodes a kinase involved in intracellular signaling, lysosomal function, and autophagy. Its mutations increase kinase activity and impair vesicle trafficking, amplifying neuronal vulnerability [161]. Another dominant gene, VPS35, involved in endosomal-lysosomal sorting, has been linked to PD through whole-exome sequencing studies [162].

In autosomal recessive forms of PD, PRKN (PARK 2) encodes Parkin, a ubiquitin E3 ligase that ensures mitochondrial quality control through autophagy of damaged mitochondria. Dysfunction in PRKN leads to mitochondrial accumulation and oxidative stress [163]. Similarly, PINK1 (PARK 6), a mitochondrial PTEN-induced kinase, works with Parkin to shield neurons from oxidative damage and apoptosis by promoting mitophagy [164]. DJ-1 (PARK7) acts as a redox-sensitive chaperone that helps protect neurons from oxidative injury, a major contributor to dopaminergic cell loss in PD [165]. Lastly, ATP13A2, another recessive PD-associated gene, is linked to lysosomal dysfunction, further underscoring the role of impaired intracellular degradation pathways in PD pathogenesis [166].

Genome-wide association studies (GWAS) have identified over 200 genetic loci linked to sporadic PD, demonstrating the polygenic nature of the disease. Current treatments for PD are primarily symptomatic, targeting dopamine synthesis or metabolism through LD, COMT inhibitors, and MAO-B inhibitors. However, gene therapy offers new avenues to address underlying pathology. Disease-modifying strategies include delivering glial cell line-derived neurotrophic factor (GDNF) to support neuronal survival, while non-disease-modifying approaches aim to enhance dopamine production by increasing the expression of enzymes like aromatic L-amino acid decarboxylase (AADC) and tyrosine hydroxylase [167]. These emerging therapies may offer more durable symptom control and fewer systemic side effects, but challenges remain in optimizing delivery mechanisms and ensuring long-term efficacy.

Leucine-rich repeat kinase 2 (LRRK2) inhibitor:* *The LRRK2 gene, identified in 2004, codes for a large protein containing serine and threonine kinase activity. It connects to the PARK8 locus, which was linked to PD back in 2002 [168,169]. Mutations in the LRRK2 gene are linked to PD, with missense mutations resulting in increased autophosphorylation and substrate phosphorylation of LRRK2. This abnormal gain in kinase activity results in lysosomal dysfunction, impaired clearance, and toxin aggregation, all contributing to PD [170]. Inhibiting LRRK2 kinase activity can mitigate the toxic effects of lysosomal dysfunction and its neurodegenerative effects. The progress in developing LRRK2 kinase inhibitors is well underway, with several compounds now in preclinical and clinical trials, targeting both central and peripheral aspects of PD [171].

Discussion

PD remains a critical public health challenge due to its progressive neurodegenerative nature and the lack of therapies that halt or reverse its progression. The global prevalence of PD is expected to double by 2040, highlighting the urgent need for disease-modifying interventions, as its economic burden already exceeds $20 billion annually in the U.S. [6, 172]. While traditional symptomatic treatments such as LD, dopamine agonists, and MAO-B inhibitors remain the mainstay of care, emerging therapies targeting neurodegeneration, neuroprotection, and neuroregeneration offer new hope.

Mitochondrial dysfunction and oxidative stress are central drivers of PD pathogenesis. Dysregulation of PGC-1α, a master regulator of mitochondrial metabolism, leads to increased neuronal vulnerability. Pioglitazone, a PPARγ agonist, has shown promise in reducing oxidative stress and improving mitochondrial function in dopamine-rich brain regions [173]. Additionally, environmental toxins such as pesticides and endogenous stressors like elevated iron levels further exacerbate oxidative stress and contribute to dopaminergic neuronal death, highlighting the need for targeted interventions [174]. Beyond mitochondrial dysfunction, neuroinflammation plays a significant role in disease progression, as pro-inflammatory cytokine release and microglial activation create a toxic feedback loop that accelerates neuronal damage [175]. Future research should explore immune-modulatory interventions, such as leveraging the JAK/STAT pathway for neuroprotection and enhancing innate and adaptive immune clearance of toxic aggregates [176,177]. These multi-modal strategies could help mitigate neurodegeneration and delay disease onset.

Current efforts in disease-modifying therapies are increasingly focused on targeting alpha-synuclein (α-Syn), the hallmark protein of PD. Approaches include reducing α-Syn aggregation, enhancing clearance through autophagy and ubiquitin-proteasome systems, and stabilizing its physiological tetrameric forms. Immunotherapies, small molecules targeting α-Syn oligomers, and gene-silencing technologies show potential in mitigating neurotoxicity and slowing disease progression [178-180]. Squalamine, a compound that displaces α-Syn from lipid membranes, demonstrates therapeutic potential by reducing its pathogenicity through inhibition of interactions with cellular structures [181].

Emerging genetic and molecular targets are also being explored as new avenues for intervention. One of the targets is the synaptic vesicle glycoprotein 2C (SV2C), which contributes to dopamine homeostasis and is being investigated for its potential in restoring dopaminergic signaling [182]. Additionally, non-receptor tyrosine kinase Abelson (c-Abl) has been implicated in oxidative stress-induced neurodegeneration, while c-Abl inhibitors, such as Nilotinib, show potential neuroprotective effects in preclinical studies. Despite these promising findings, challenges remain with blood-brain barrier permeability and optimal dosing, ultimately requiring further investigation through clinical trials [183,184].

As the underlying neurodegeneration progresses, regenerative strategies are being explored to replace lost dopaminergic neurons and restore functional neural networks. Cell-based therapies such as bemdaneprocel, a stem cell-derived approach, have demonstrated safety and motor function improvements in early trials, offering a promising avenue for long-term neuronal restoration [185]. Beyond cell-based regeneration, multi-modal approaches integrating genetic, molecular, and regenerative pathways could provide more comprehensive and long-lasting solutions for PD patients. The convergence of these therapeutic strategies will play a crucial role in slowing disease progression, improving patient outcomes, and ultimately achieving neurorestoration.

## Conclusions

This paper explores both current and emerging therapies for PD, focusing on dopaminergic and non-dopaminergic treatments. LD remains the gold standard for PD management, often combined with DDC inhibitors (CD, benserazide) to enhance its bioavailability and COMT inhibitors (entacapone, tolcapone) to prolong its effects. DAs such as pramipexole and ropinirole provide symptomatic relief, while MAO-B inhibitors (selegiline, rasagiline, safinamide) slow dopamine metabolism and offer potential neuroprotection. Despite their efficacy, long-term LD use is associated with motor complications, prompting the development of novel therapies such as adenosine A2A antagonists (istradefylline), which reduce OFF episodes, and GLP-1RAs, which show neuroprotective potential. Additionally, gene therapy strategies targeting LRRK2 (BIIB122) and lysosomal dysfunction (ambroxol, buntanetap) offer promising disease-modifying potential. Other innovative approaches, including cell-based regenerative therapies (bemdaneprocel), novel drug delivery systems, and gastrointestinal-focused treatments (*Lactobacillus acidophilus*), aim to address both motor and non-motor symptoms of PD. While these novel therapies hold great promise, large-scale, randomized phase 3 and phase 4 trials are needed to confirm their efficacy and long-term benefits.

Significant challenges remain regarding the management of PD, including ensuring the safety and efficacy of novel treatments, refining delivery mechanisms, and understanding their long-term impact. Addressing these gaps will require collaborative efforts among researchers, clinicians, and pharmaceutical companies to accelerate the translation of experimental therapies into clinical practice. Advancing the understanding of PD’s complex pathophysiology and refining innovative treatments has the potential to lead to transformative solutions that extend beyond symptom management to modifying the disease course, ultimately providing renewed hope to patients and their families.
